# Mesalamine-Induced Myocarditis and Coronary Vasculitis in a Pediatric Ulcerative Colitis Patient: A Case Report

**DOI:** 10.1155/2011/524364

**Published:** 2011-12-21

**Authors:** Elimarys Perez-Colon, Gul H. Dadlani, Ivan Wilmot, Michelle Miller

**Affiliations:** ^1^Department of Pediatrics, University of South Florida, 2 Tampa General Circle, Tampa, FL 33606, USA; ^2^All Children’s Hospital Heart Institute, Outpatient Care Center, 2nd Floor, 501 6th Street South Saint. Petersburg, FL 33701, USA

## Abstract

Mesalamine-containing products are often a first-line treatment for ulcerative colitis. Severe adverse reactions to these products, including cardiovascular toxicity, are rarely seen in pediatric patients. We present a case of a 16-year-old boy with ulcerative colitis treated with Asacol, a mesalamine-containing product, who developed sudden onset chest pain after four weeks on therapy. Serial electrocardiograms showed nonspecific ST segment changes, an echocardiogram showed mildly decreased left ventricular systolic function with mild to moderate left ventricular dilation and coronary ectasia, and his troponins were elevated. Following Asacol discontinuation, his chest pain resolved, troponins were trending towards normal, left ventricular systolic function normalized, and coronary ectasia improved within 24 hours suggesting an Asacol-associated severe drug reaction. Mesalamine-induced cardiovascular toxicity, although rare, may represent a life-threatening disorder. Therefore, every patient presenting with acute chest pain should receive a workup to rule out this rare drug-induced disorder.

## 1. Introduction

Mesalamine-(5-aminosalicylic acid) containing products are a well-known treatment for inflammatory bowel disease, often as first line. Mesalamine's mechanism of action is not completely understood, but seems to reduce colonic inflammation topically by inhibiting the cyclooxygenase pathway and the *γ*-form of peroxisomal proliferator-activated receptors (PPAR-*γ*) signaling pathway. Adverse reactions to mesalamine are uncommon and include mostly gastrointestinal upset and headaches [1]. Rare drug reactions to mesalamine have been described in the literature and include pancreatitis, blood dyscrasias, cardiovascular problems, and interstitial nephritis [2]. Although rare, pericarditis, myocarditis, vasculitis, and left ventricular dysfunction have been described with mesalamine therapy [3–8]. We present a case of myocarditis with coronary vasculitis as a reaction to a mesalamine product, Asacol.

## 2. Case Presentation

This is a case of a 16-year-old Hispanic male without significant past medical history admitted for evaluation following 3-month history of abdominal pain and bloody diarrhea. Outpatient pediatric gastroenterology evaluation was negative for infectious causes, and due to ongoing symptoms, he was admitted for further evaluation and treatment. Colonoscopy performed on admission showed pancolitis with friable tissue, and biopsies were taken confirming ulcerative colitis (UC) as the diagnosis. He was started on methylprednisolone 0.5 mg/kg/dose twice a day and Asacol 400 mg three times a day (30 mg/kg/day). His symptoms persisted, and on hospital day number four his Asacol dose was increased to 800 mg three times per day (60 mg/kg/day). His abdominal pain and bloody diarrhea started to improve on the new regimen up until hospital day nine when he developed worsening of symptoms with an acute episode of bright red blood per rectum. At that point, Azathioprine 4 mg/kg/day was added and the patient was started on total parenteral nutrition. On hospital day number ten, he developed a right upper arm superficial thrombosis with superimposed methicillin-sensitive *Staphylococcus* thrombophlebitis. Hematology was involved, and he was started on anticoagulation therapy with low-molecular-weight heparin (LMWH) and cefazolin. A hypercoagulable workup, including prothrombin 2021A, factor 5 Leiden mutation, homocysteine, anticardiolipin antibody, protein C, protein S, lupus anticoagulant, and Antithrombin III, was obtained and resulted negative. On hospital day eleven, he was noted to have intermittent systemic hypertension. Pediatric nephrology was consulted, and a renal ultrasound was noted to be normal without renal artery thrombosis or stenosis. His renal function was within normal limits, and he was started on nifedipine as needed for systolic blood pressure greater than 150 mmHg. His elevated blood pressure was thought to be secondary to steroids. Over the course of the hospitalization, his platelets began to drop and on hospital day number fifteen he had a large episode of hematochezia and some epixtasis. At that point, the LMWH was stopped and he received packed red blood cells and platelet transfusions. Due to persistent thrombocytopenia with poor response to platelet transfusion, he underwent a bone marrow aspirate that showed cellular marrow with active hematopoiesis. He was diagnosed with idiopathic thrombocytopenic purpura (ITP) with positive antiplatelets antibodies and was treated with intravenous immunoglobulin (IVIG), 1 gram/kilogram/day, without major events. 

**Figure 1:**
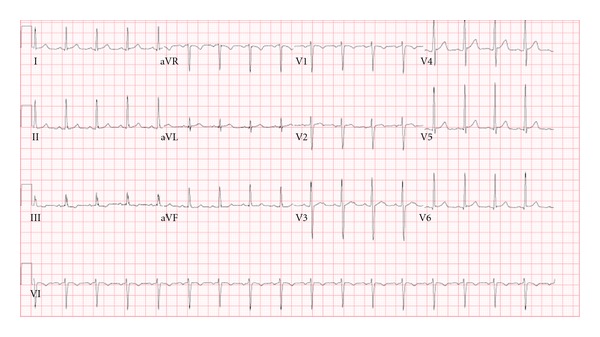
Electrocardiogram on day of chest pain onset: sinus tachycardia with no ST segment changes.

On hospital day number twenty-nine, he developed acute chest pain described as throbbing pain located in the middle of his chest without radiation. His physical exam was unremarkable except for elevated blood pressure at 152/91 mmHg and tachycardia with heart rate of 109. His electrocardiogram showed sinus tachycardia without ST segment changes (Figure 1). His troponin-I was elevated and peaked at 0.67 ng/mL, and cardiology was consulted. 

**Figure 2:**
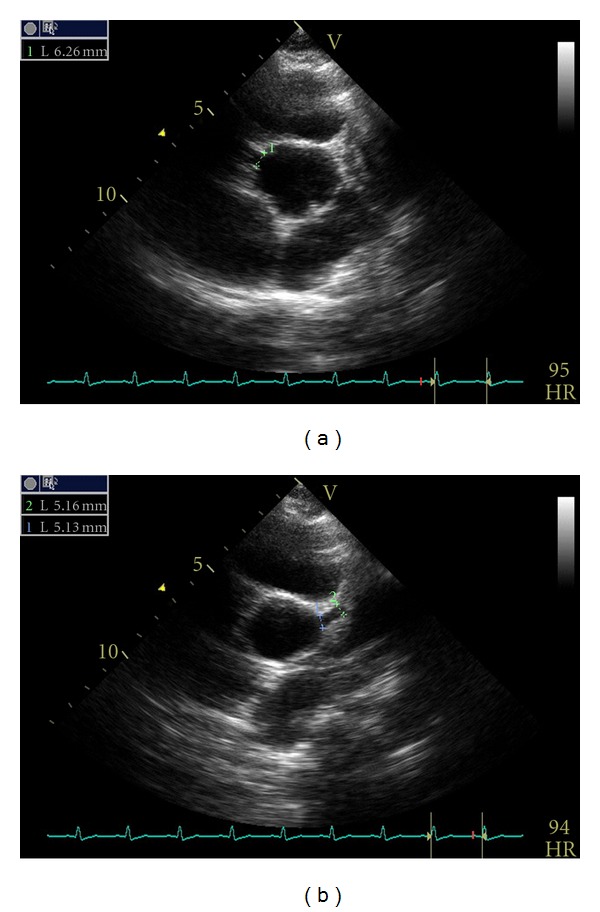
Coronary artery ectasia. Right coronary artery diameter measuring 6 mm (a) and left coronary artery diameter measuring 5 mm (b).

**Figure 3:**
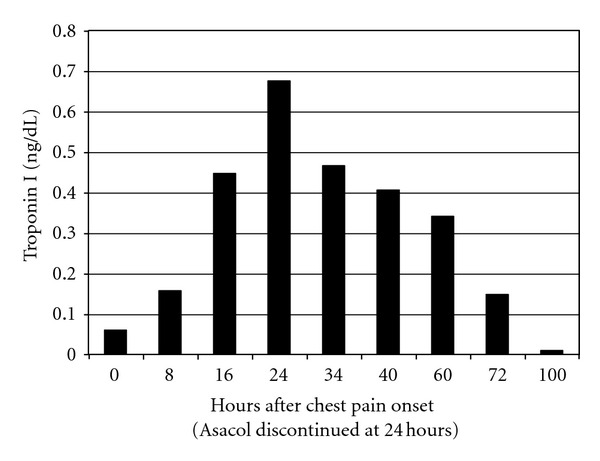
Cardiac enzymes trend on and off Asacol.

A transthoracic echocardiogram revealed mild to moderate dilated left ventricle, mild left ventricular systolic dysfunction with an ejection fraction (EF) of 48%, and bilateral coronary artery ectasia (Figure 2). His right coronary artery measured 6 mm in diameter, and the left main coronary artery was 5 mm in diameter. He was noted to have normal intracardiac anatomy. He was transferred to the pediatric intensive care unit for close cardiovascular monitoring. A decision to discontinue Asacol was made on hospital day thirty, since it was thought to be the most likely etiology of his symptoms. After Asacol discontinuation, his chest pain resolved and his troponins were noted to trend towards normal (Figure 3).

**Figure 4:**
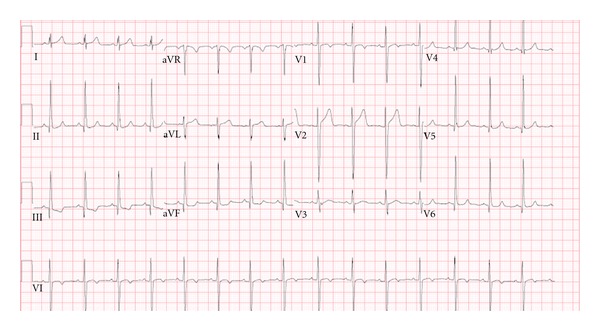
Electrocardiogram 6 hours off Asacol: normal sinus rhythm and nonspecific ST changes.

**Figure 5:**
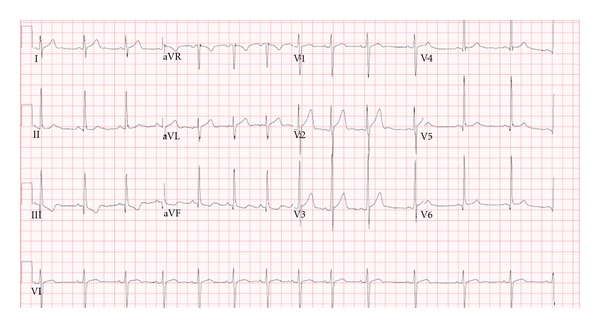
Electrocardiogram 7 days off Asacol: normal sinus rhythm and T wave inversion lead III.

An electrocardiogram on hospital day one off Asacol showed normal sinus rhythm with nonspecific ST segment changes in the inferior leads (Figure 4). A repeated echocardiogram showed improved left ventricular function with an EF of 59% and improved coronary artery dilation after two days off Asacol. An echocardiogram performed three days following Asacol discontinuation showed mild dilatation of the left ventricle with normal systolic function (EF 56%) and improved coronary artery dilation, although there was mild persistent proximal dilation. His EKG showed normal sinus rhythm with nonspecific ST segments changes. His troponin-I continued to trend down, and he was discharged home. He was seen on followup after a week of Asacol discontinuation, and his electrocardiogram showed normal sinus rhythm with T wave inversion in lead III (Figure 5). His echocardiogram showed mildly dilated left ventricle with an ejection fraction of 66% and mildly improved proximal dilation of the coronary arteries. His right coronary artery measured 3.8 mm, and his left main coronary artery measured 4.8 mm.

## 3. Discussion

Cardiac complications in patients with inflammatory bowel disease may represent a rare extraintestinal manifestation or drug-related side effect [3, 7]. Cardiac manifestations of ulcerative colitis (UC) are uncommon and include pericarditis, pericardial effusion, and few cases of pericardiac tamponade. UC-associated pericarditis is a diagnosis of exclusion after metabolic causes, infection, malignancy, and connective tissue disorders have been ruled out [9]. Mesalamine-(5-aminosalicylic acid) induced cardiovascular toxicity, including pericarditis, myocarditis, vasculitis, and left ventricular dysfunction, is a rare complication that has been described in the literature [3–8]. Mesalamine's mechanism of action is not fully understood and includes inhibition of the cyclooxygenase pathway and therefore decreased prostaglandins synthesis and inhibition of the PPAR-*γ* signaling pathway resulting in decreased activity of nuclear factor *κ*B and as a consequence decreased inflammation in the colon [3, 10]. The specific mechanism for mesalamine-induced cardiovascular toxicity is not completely understood but is thought to be a hypersensitivity reaction rather than a cytotoxic effect. A proposed mechanism is humoral-mediated hypersensitivity in which antibodies formed against mesalamine cross-react with cardiac tissue causing inflammation [8]. Most cases of mesalamine-induced cardiovascular toxicity occur 2–4 weeks after the initial exposure to the drug, although presentation may be delayed in the setting of concomitant steroid administration [8]. Resolution of symptoms has occurred in all reported cases within one week after drug discontinuation. 

In our case, the patient's onset and resolution of symptoms and signs are similar to those reported in the literature. Within twenty-four hours after Asacol discontinuation, our patient had normalized left ventricular function (Table 1), making mesalamine-induced myocarditis the most likely diagnosis. Coronary artery ectasia, seen in our patient, has been previously described in a similar case in the literature and may represent a vasculitis type inflammation induced by mesalamine by a mechanism similar to the one described above. Although improved after Asacol discontinuation, mild proximal coronary ectasia persisted one week following Asacol discontinuation. We will continue to follow this finding for further correlation. 

Our patient also received IVIG during his hospital stay for ITP, and there are several cases of non-ST elevation as well as ST elevation myocardial infarction described in the adult literature in patients with prior risk factors for cardiovascular disease (i.e., hypertension, diabetes, and coronary artery disease) after a first cycle of IVIG; this is likely secondary to hyperviscosity and complement activation causing occlusion of vessels already narrowed by atherosclerosis [11–13]. Our patient did not have any risk factor for cardiovascular disease and his chest pain presented a week after the IVIG infusion. There are also no reported cases in the pediatric population making IVIG a very unlikely suspect. 

Mesalamine-induced cardiovascular toxicity, although rare, may represent a life-threatening disorder that requires immediate discontinuation of the mesalamine-containing product and adequate supportive treatment. It is suggested that every patient, on mesalamine, presenting with acute chest pain, shortness of breath, or any additional cardiovascular concern undergoes an electrocardiogram, cardiac enzymes, and an echocardiogram to rule out this rare drug-induced disorder.

## Figures and Tables

**Table 1:** Correlation of symptoms progression and diagnostic data on and off Asacol.

	On Asacol day 29	Off Asacol day 1	Off Asacol day 2	Off Asacol day 3	Off Asacol day 7
Symptoms	Chest pain	Chest pain	None	None	None

Troponin	0.157 ng/mL	0.466 ng/L	0.341 ng/mL	0.148 ng/mL	<0.010 ng/mL
	0.447 ng/mL	0.406 ng/mL			
	0.67 ng/mL				

EKG	Sinus Tachycardia With no ST segment Changes (Figure 1)	Normal sinus rhythm with nonspecific ST changes in inferior leads (Figure 4)	Normal sinus rhythm with nonspecific ST changes	Normal sinus rhythm with sinus arrhythmia and no ST segment Changes	Normal sinus rhythm with sinus arrhythmia and T wave inversion in lead III (Figure 5)

ECHO	Mild-moderate dilated LV	Mildly dilated LV	Mildly dilated LV	Mildly dilated LV	Normal LV
	EF at 48%	EF at 65%	EF at 59%	EF at 56%	EF at 66%
	Dilated coronary arteries RCA 6 mm (Z ≥ +4) LCA 5 mm (Z = +2).	Proximal dilation of coronary arteries	Proximal dilation of coronary arteries RCA 6.1 mm	Proximal dilation of coronary arteries RCA 4.5 mm LCA 5 mm LDA 3.4 mm	Proximal dilation of coronary arteries RCA 3.8 mm LCA 4.8 mm LDA 3.6 mm
